# Evaluation of a rapid Loop Mediated Isothermal Amplification (LAMP) test for the laboratory diagnosis of sexually transmitted infections

**DOI:** 10.1371/journal.pone.0298398

**Published:** 2024-03-21

**Authors:** Martina Brandolini, Laura Grumiro, Patrizia Farabegoli, Giorgio Dirani, Silvia Zannoli, Irene Zaghi, Massimiliano Guerra, Francesca Taddei, Giulia Gatti, Anna Marzucco, Maria Sofia Montanari, Alessandra Mistral De Pascali, Simona Semprini, Monica Cricca, Vittorio Sambri

**Affiliations:** 1 Unit of Microbiology, The Greater Romagna Area Hub Laboratory, Cesena, Italy; 2 Department Medical and Surgical Sciences (DIMEC)—Alma Mater Studiorum, University of Bologna, Bologna, Italy; 3 Unit of Infectious Diseases, Santa Maria delle Croci Hospital, Ravenna, Italy; Hawassa University College of Medicine and Health Sciences, ETHIOPIA

## Abstract

Sexually transmitted infections (STIs) have seen a considerable increase in the last years and given the health burden they may represent from both a personal and community perspective, they require surveillance and prevention programmes based on a timely and decentralized diagnosis. In this context, user-friendly rapid molecular tests may represent a good trade-off between diagnostic accuracy, accessibility and affordability. In this study we evaluated the diagnostic performance of a new real-time LAMP (Loop Mediated Isothermal Amplification) method for the rapid detection and differentiation of 7 major sexually transmissible pathogens by analysing real clinical samples (genital and extra-genital matrices) from individuals with suspected STIs. The assay showed good overall diagnostic performances in terms of sensitivity, specificity and concordance with a gold-standard PCR-based molecular method. This assay, not requiring specialised laboratory technicians or expensive instrumentation, but nonetheless capable of guaranteeing accurate results, is within the reach of outpatient settings, obstetrics, and gynaecology clinic, hence ensuring on-field access to early diagnosis.

## Introduction

Sexually transmitted infections (STIs) are a major public health concern worldwide and have a profound impact on sexual and reproductive health, with more than one million STIs acquired every day [1]. To date, more than 30 pathogens, comprehending bacteria, viruses and protozoan can be made accountable of STIs. Despite most STIs are not fatal per se, they nonetheless constitute a non-negligible health problem, increasing the risk of HIV acquisition, cervical, anal or oral cancer, reproductive issues, potentially resulting in pelvic inflammatory disease and infertility [2, 3], and congenital deformities or even stillbirth and neonatal death secondary to mother-to-child transmission [4–7]. Free sexual behaviours, decreased condom use, increased number of sexual partners, mixed with the misconception that HIV pre-exposure prophylaxis is sufficient to protect from STIs transmission, have surely contributed to a significant increase in STIs prevalence. Surveillance programmes have been implemented locally, nationally, and internationally to monitor STIs incidence. In Italy, two STIs sentinel surveillance systems are in place, both coordinated by the Italian Institute of Public Health. Clinical Surveillance, active since 1991, is responsible of notifying symptomatic subjects with a clinical diagnosis of STIs. Laboratory Surveillance, active since 2009, on the other hands, deals with the notification of new cases of infection with *Chlamydia trachomatis*, *Trichomonas vaginalis* and *Neisseria gonorrhoeae* in people who undergo laboratory tests for STI, regardless of specific symptoms. The integration of the results of these two programs, together with socio-demographic, behavioural habits, and health data, allows to measure relative frequency and temporal trends of STIs over time, as well as to evaluate the associated risk factors. From 2005 to 2019, reports of clinically overt STIs increased by 41.8% compared to the period 1991–2004, with *C*. *trachomatis* showing a higher prevalence than *T*. *vaginalis* and *N*. *gonorrhoeae*, as attested by microbiological surveillance [8]. European and global trends reflect the Italian situation [9–11].

Early but also accurate diagnosis and identification of asymptomatic carriers are imperative and represent the basis for surveillance and prevention programmes implementation [10]. In this context, syndromic management of STIs, solely based upon observation and interpretation of clinical symptoms, without the need of further laboratory confirmation of the putatively responsible pathogen, is often misleading due to the generally asymptomatic course of these infections or the overlap of clinical symptoms between different STI-causing pathogens [12, 13]. STIs laboratory confirmation therefore represents a cornerstone not only for etiological diagnosis, but, in a broader perspective, also for screening and surveillance, allowing the identification of asymptomatic infections and hence the determination of the real spread of STIs [14]. Molecular methods represent the gold standard for STIs diagnosis due to their accuracy and sensitivity [15]. These perspectives and necessities call for the development of cost-effective, user-friendly and broadly available molecular assays easily deployed in obstetrics and gynaecology clinic, emphasising the importance of improved access to early and accurate detection aimed at population surveillance and pathogen circulation mapping [16].

The aim of this study is to evaluate the diagnostic performance of a new real-time LAMP (Loop Mediated Isothermal Amplification) method for the rapid detection and differentiation of 7 major sexually transmissible pathogens, comprehending bacterial STIs (caused by *Chlamydia trachomatis*, *Neisseria gonorrhoeae* and non-chlamydial non-gonococcal bacteria, such as *Mycoplasma genitalium*, *Mycoplasma hominis*, *Ureaplasma urealyticum* and *Ureaplasma parvum*), and protozoa (*Trichomonas vaginalis*). The test was run on an easy-to-use and low-cost instrument, which enables a real-time monitoring of the fluorescent-tagged probe signal, with a sample-to-results time of approximately 70 minutes, including a fast and easy DNA extraction and the following amplification reaction. Enbiotech STI panel (Enbiotech, Palermo, Italy) results were compared with Allplex™ STI Essential Assay (Seegene, Seoul, South Korea). The present work was carried out in the context of IVDR (In Vitro Diagnostic Regulation) validation of Enbiotech STI panel.

## Materials and methods

### Study design, population, and sample collection

The study was conducted by analysing retrospectively 557 anonymised samples collected between February and May 2023 from subjects who reported symptoms suggestive of a sexually transmissible infection or sought medical attendance after unprotected sexual intercourses in hospitals of the Romagna area (Forlì-Cesena, Rimini and Ravenna provinces), north-eastern Italy. A total of 121 urine samples, 25 semen samples, 334 vaginal swabs, 27 anal swabs, 39 urethral swabs, 9 oropharyngeal swabs and 2 placental swabs were included. All swabs were collected using FLOQswabs (Copan, Brescia, Italy). The samples were conferred to the Unit of Microbiology, Greater Romagna Area Hub Laboratory, Cesena, Italy, for routine molecular diagnosis of STIs employing Allplex™ STI Essential Assay and results were reported as answer to a clinical suspicion. Before being included in this study, all samples underwent an anonymization procedure, in order to adhere to the regulations issued by the Romagna Local Health Authority Ethical Board. No information that could identify individual participants was accessed. As such, based on the local regulation on the use of archived anonymised samples (protocol code AVR-PPC P09, rev.2, based on Burnett et al., 2007 [17]), ethical approval and informed written consent from participants was not necessary.

The project was divided in two phases: in the first phase we evaluated the performance of the LAMP mix and primers/probe sets produced by Enbiotech by analysing DNA eluates obtained with an automated extraction method (STARMag 96 X 4 Universal Cartridge Kit). The second phase of the project aimed at the evaluation of the entire point-of-need-designed simplified workflow, which includes a non-automated thermal/chemical extraction step followed by LAMP amplification. In both phases, obtained results were compared with the ones obtained with the gold standard extraction (STARMag 96 X 4 Universal Cartridge Kit) and amplification methods (Allplex™ STI Essential Assay). For the following discussion of the results, consider the different Limits of Detection (LoDs) of the two assays, as reported in Table 1.

**Table 1:** Limits of Detection (LoDs), expressed in genomic copies per reaction for the different targets of the two molecular assays employed, as declared by the manufacturers.

	Detection limit (genomic copies/reaction)	
Organism	Allplex™ STI Essential Assay (Seegene Inc)	Enbiotech STI Panel (Enbiotech)
***Ureaplasma urealyticum***	10^3^	39
***Ureaplasma parvum***	10^5^	42
***Neisseria gonorrhoeae***	10^1^	6.41
***Mycoplasma hominis***	10^2^	40
***Mycoplasma genitalium***	5x10^1^	42
***Chlamydia trachomatis***	10^1^	27
***Trichimonas vaginalis***	10^1^	26

### Automated extraction and Allplex™ STI Essential Assay multiplex PCR

All samples were extracted using STARMag 96 X 4 Universal Cartridge Kit and DNA eluates were subsequently amplified using Allplex™ STI Essential Assay, a multiplex real-time quantitative PCR (Polymerase Chain Reaction) assay, which enables the rapid amplification and detection of 7 bacterial and protozoan species responsible of STIs (*C*. *trachomatis*, *T*. *vaginalis*, *N*. *gonorrhoeae*, *M*. *genitalium*, *M*. *hominis*, *U*. *urealyticum* and *U*. *parvum*). Both extraction and PCR setup were performed according to the manufacturer instructions [18, 19] on a STARlet automated workstation (Seegene). PCR amplification was run on a CFX96 real-time thermal cycler (Bio-Rad, Feldkirchen, Germany).

### Automated extraction and LAMP mix and primers/probes sets evaluation

**Fig 1:**
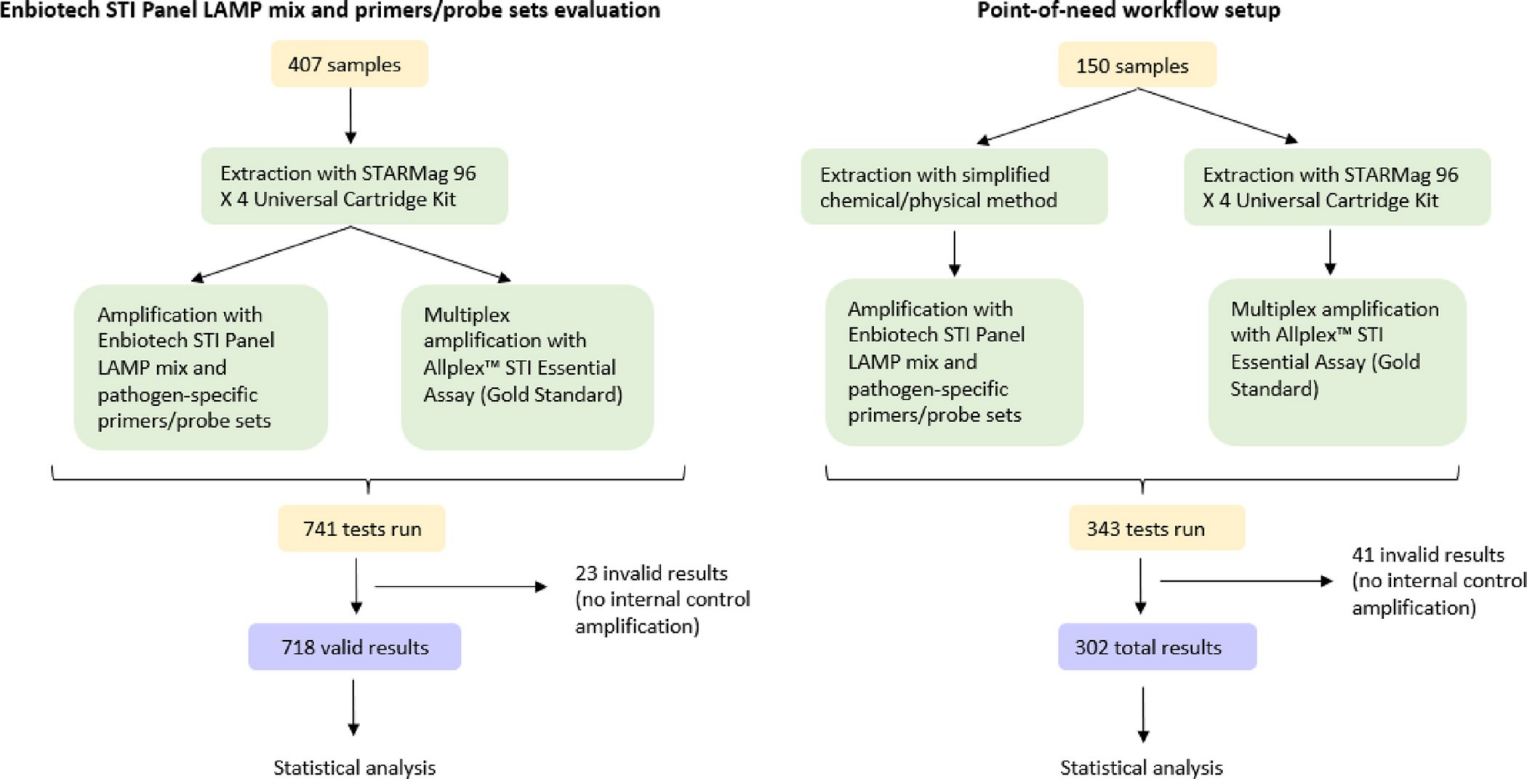
Graphical representation of Enbiotech STI Panel primers/probe sets evaluation and point-of-need workflow setup phases. The two distinct workflows employed, firstly, for the evaluation of the Enbiotech STI Panel LAMP pathogen-specific primers/probe sets, and secondly for the set-up of the point-of-need assay are represented.

Performance of LAMP mix and primers/probes sets was evaluated by analysing DNA eluates obtained from automated extraction with STARMag 96 X 4 Universal Cartridge Kit. LAMP reactions were set up according to the manufacturer instruction. In brief, 19 μL of LAMP mix were added to strips containing lyophilised pathogen-specific primers and probe, followed by 30 μL of mineral oil (to reduce non-specific amplification) [20, 21]; 6 μL of extracted DNA were then pipetted directly into the mix. The reaction was run on an ICGene instrument (Enbiotech) at 60°C for 60 minutes. Fluorophore-tagged probe signals were automatically acquired. For every reaction, the amplification of an endogenous internal control (beta-actin) was also monitored in order to confirm adequacy of collected samples, presence of amplifiable DNA and absence of inhibitory substances in extracted eluates. Thereby obtained results were compared with results obtained by processing samples with the gold standard extraction and amplification methods. Enbiotech STI Panel LAMP mix and primers/probe sets evaluation workflow is schematically summarised in Fig 1.

### Point-of-need workflow setup

In order to evaluate the possibility of using this kit as a point-of-need test, a rapid, easy and non-automated crude DNA extraction method was evaluated. Samples were briefly vortexed and 500 μL (for semen samples, vaginal swabs, anal swabs, urethral swabs and oropharyngeal swabs) or 1 mL (urine samples) were transferred to 1.5 mL of extraction buffer, vortexed and heated at 95°C for 10 minutes. LAMP reaction setup, amplification and data acquisition were performed as previously described. Thereby obtained results were compared with results obtained by processing samples with the gold standard extraction and amplification methods. Point-of-need workflow is schematically summarised in Fig 1.

### Statistical analysis

Statistical analysis was carried out using OriginPro 8.5 (OriginLab Corporation, Northampton, MA, USA). To evaluate Enbiotech STI Panel analytical performance compared to Allplex™ STI Essential Assay sensitivity, specificity, positive predictive value, negative predictive value, test accuracy and Cohen’s Kappa were calculated separately for every tested pathogen. The corresponding two-tailed 95% score (Wilson) confidence intervals (CI) were also estimated.

## Results

### LAMP mix and primers/probe sets evaluation

**Fig 2:**
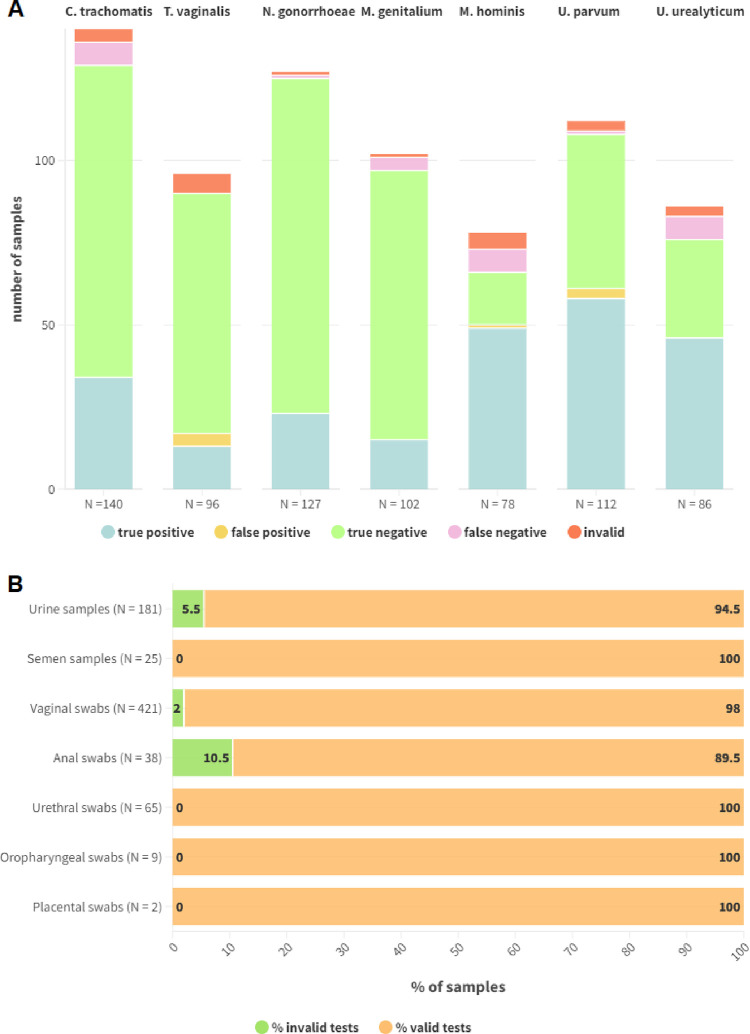
Graphical representation of the results obtained for Enbiotech STI Panel LAMP mix and primers/probe sets evaluation. In panel A, true positive, false positive, true negative, false negative and invalid results are separately shown for each tested pathogen; the total number of samples tested with every pathogen-specific primers/probe set are specified below every stacked column. In panel B percentages of valid and invalid results are shown separately for every matrix; the total number of tested samples for every matrix in reported between brackets.

A total of 407 samples previously extracted with STARMag 96 X 4 Universal Cartridge Kit were tested with Enbiotech STI Panel to evaluate LAMP mix and primers/probe sets sensitivity and specificity. Of these, 106 (26%) were urine samples, 19 (5%) semen samples, 230 (57%) vaginal swabs, 14 (3%) anal swabs, 30 (7%) urethral swabs, 6 (1%) oropharyngeal swabs and 2 (0.5%) placental swabs. A total of 101 samples (25%) were negative for all the tested pathogens, 167 (41%) were positive for one pathogen, while 139 (34%) were coinfections: 102 (25%) two-pathogen coinfections, 27 (7%) three-pathogen, 8 (2%) four-pathogen and 2 (0.5%) five-pathogen. Some samples were tested with more than one sets of primers resulting in a total of 741 tests run: 181 (24%) on urines, 25 (3%) on semen, 421 (57%) on vaginal swabs, 38 (5%) on anal swabs, 65 (9%) on urethral swabs, 9 (1%) on oropharyngeal swabs and 2 (0.5%) on placental swabs. Overall, 140 samples were tested for *C*. *trachomatis*, 96 for *T*. *vaginalis*, 127 for *N*. *gonorrhoeae*, 102 for *M*. *genitalium*, 78 for *M*. *hominis*, 112 for *U*. *parvum* and 86 for *U*. *urealyticum* using Enbiotech STI Panel pathogen-specific primers/probe sets. Upon testing 23 samples gave an invalid result due to a failed internal control amplification (23/741 total tests run, 3.1%), represented by 10 urines (10/181, 5.5%) 9 vaginal swabs (9/421, 2.1%, and 4 anal swabs (4/38, 10.5%). Percentages of invalid tests were calculated considering the total number of tests run on that matrix. Overall, 8 samples (8/741, 1%) gave a false positive result (4 with *T*. *vaginalis* primers, 1 with *M*. *hominis* primers and 3 with *U*. *urealyticum* primers), while 27 (27/741, 4%) were false negative (7 with *C*. *trachomatis* primers, 1 with *N*. *gonorrhoeae* primers, 4 with *M*. *genitalium* primers, 1 with *U*. *parvum* primers, and 7 with *U*. *urealyticum* primers) due to the low pathogen load (Allplex™ STI Essential Assay Ct (Cycle threshold) > 27). Results for STI Panel LAMP mix and primers/probe sets evaluation is shown in Fig 2.

Results obtained from amplification with Enbiotech STI Panel of automatedly extracted samples were compared with Allplex™ STI Essential Assay performed on the same extracts. Sensitivity, specificity, positive predictive value (PPV), negative predictive value (NPV), test accuracy and Cohen’s Kappa were separately calculated for every set of primers/probe based on the number of valid tests for every tested pathogen. Sensitivity ranged from 79.0% for *M*. *genitalium* to 100% for *T*. *vaginalis*, specificity ranged from 94.0% for *U*. *parvum* to 100% *C*. *trachomatis*, *N*. *gonorrhoeae*, *M*. *genitalium* and *U*. *urealyticum*. PPV ranged from 76.5% for *T*. *vaginalis* to 100% for *C*. *trachomatis*, *N*. *gonorrhoeae*, *M*. *genitalium*, and *U*. *urealyticum*, while NPV ranged from 69.6% for *M*. *hominis* to 100% for *T*. *vaginalis*. Overall, calculated test accuracy was above 89% for all tested primers/probes sets. Calculated Cohen’s Kappa highlighted a good to really good agreement between the two amplifications methods performed on automatedly extracted samples, ranging from 0.73 for *M*. *hominis* to 0.97 for *N*. *gonorrhoeae*. Detailed information regarding test sensitivity, specificity, PPV, NPV, accuracy and Cohen’s Kappa are reported in Table 2.

**Table 2:** Results of the statistical analysis for the evaluation of Enbiotech STI Panel pathogen-specific primers/probe sets. Data obtained from the amplification of automatedly extracted samples (STARMag 96 X 4 Universal Cartridge Kit, Seegene) with Allplex™ STI Essential Assay (Seegene) were compared with the ones obtained from the analysis of the same extracts with Enbiotech STI Panel pathogen-specific primers/probe sets. For every indicator its respective 95% confidence interval was calculated.

	Sensitivity	Specificity	PPV	NPV	Accuracy	Cohen’s Kappa
***C*. *trachomatis***	82.9%	100.0%	100.0%	93.1%	94.9%	0.87
	67.94–92.85	96.19–100.00	89.72–100.00	86.37–97.20	89.68–97.91	0.78–0.96
***T*. *vaginalis***	100.0%	94.8%	76.5%	100.0%	95.6%	0.84
	75.29–100.00	87.23–98.57	50.10–93.19	95.07–100.00	89.01–98.78	0.69–0.99
***N*. *gonorrhoeae***	95.8%	100.0%	100.0%	99.0%	99.2%	0.97
	78.88–99.89	96.45–100.00	85.18–100.00	94.71–99.98	95.66–99.98	0.92–1.00
***M*. *genitalium***	79.0%	100.0%	100.0%	95.4%	96.0%	0.86
	54.43–93.95	95.60–100.00	78.20–100.00	88.52–98.72	90.17–98.91	0.72–0.99
***M*. *hominis***	87.5%	94.1%	98.0%	69.6%	89.0%	0.73
	75.93–94.82	71.31–99.85	89.35–99.95	47.08–86.79	79.54–95.15	0.55–0.90
***U*. *parvum***	98.3%	94.0%	95.1%	97.9%	96.3%	0.93
	90.91–99.96	83.45–98.75	86.29–98.97	88.93–99.95	90.87–98.99	0.85–1.00
***U*. *urealyticum***	86.8%	100.0%	100.00%	81.08%	91.57%	0.82
	74.66–94.52	88.43–100.00	92.29–100.00	64.84–92.04	83.39–96.54	0.70–0.95

CI = Confidence Interval, PPV = Positive Predictive Value, NPV = Negative Predictive Value.

### Point-of-need workflow

**Fig 3:**
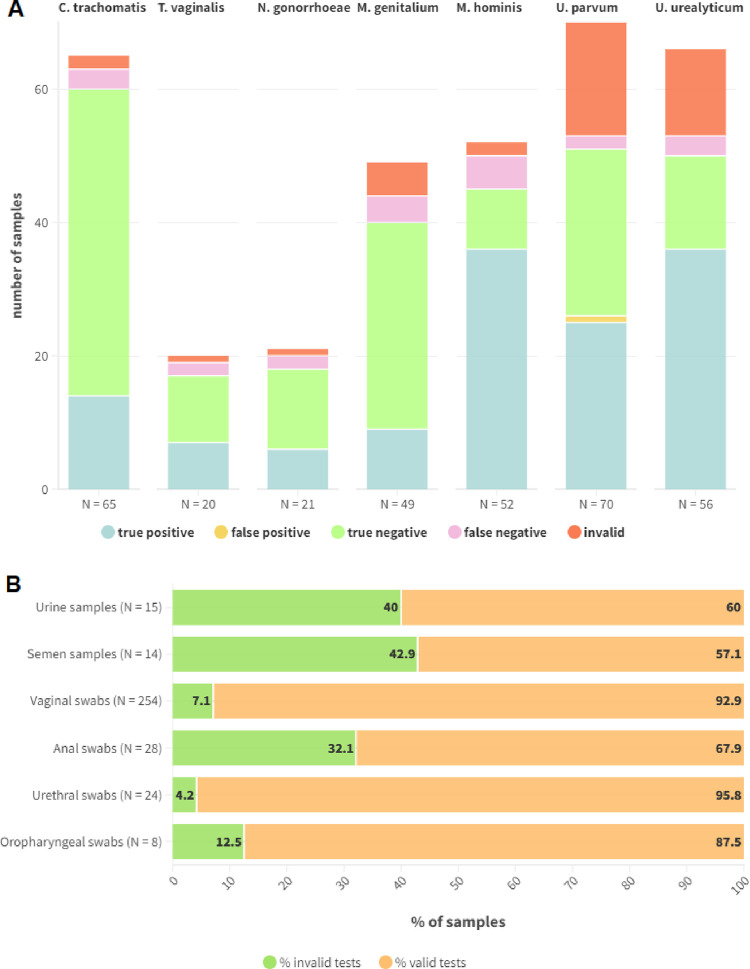
Graphical representation of the results obtained for point-of-need workflow setup. In panel A, true positive, false positive, true negative, false negative and invalid results are separately shown for each tested pathogen; the total number of samples tested with every pathogen-specific primers/probe set are specified below every stacked column. In panel B percentages of valid and invalid results are shown separately for every matrix; the total number of tested samples for every matrix in reported between brackets.

To evaluate the performance of the entire point-of-need workflow, 150 samples were extracted with the aforementioned simplified protocol and then tested with Enbiotech STI Panel LAMP mix and primers/probe sets. Of these 15 (10%) were urine samples, 6 (4%) semen samples, 104 (69%) vaginal swabs, 13 (9%) anal swabs, 9 (6%) urethral swabs, and 3 (2%) oropharyngeal swabs. Total number of tested samples at this stage was limited by the availability of sufficient sample volumes residual from routine diagnostic activities. A total of 11 samples (7%) were negative for all the tested pathogens, 80 (53%) were positive for one pathogen, while 59 (39%) were coinfections: 47 (31%) two-pathogen coinfections, 7 (5%) three-pathogen, 3 (2%) four-pathogen and 2 (1%) five-pathogen. Some samples were tested with more than one sets of primers resulting in a total of 343 tests run: 15 (4%) on urines, 14 (4%) on semen, 254 (74%) on vaginal swabs, 28 (8%) on anal swabs, 24 (7%) on urethral swabs, and 8 (2%) on oropharyngeal swabs. Overall, 65 samples were tested for *C*. *trachomatis*, 20 for *T*. *vaginalis*, 21 for *N*. *gonorrhoeae*, 49 for *M*. *genitalium*, 52 for *M*. *hominis*, 70 for *U*. *parvum* and 66 for *U*. *urealyticum*. In 41 samples internal control was not amplified and detected correctly, so they were considered invalid (41/343 of total tests run, 12%); of these 6 were urine samples (6/15, 40%), 6 semen samples (6/14, 42.9%), 18 vaginal swabs (18/254, 7.1%), 9 anal swabs (9/28, 32.1%), 1 urethral swab (1/24, 4.2%), and 1 oropharyngeal swab (1/8, 12.5%). Percentages of invalid tests were calculated considering the total number of tests run on that matrix. Overall, 1 sample (1/343, 0.3%) gave a false positive result with *U*. *parvum* primers, while 21 (21/343, 6%) were false negative (3 with *C*. *trachomatis* primers, 2 with *T*. *vaginalis* primers, 2 with *N*. *gonorrhoeae* primers, 4 with *M*. *genitalium* primers, 5 with *M*. *hominis* primers, 2 with *U*. *parvum* primers, and 3 with *U*. *urealyticum* primers) due to the low pathogen load (Allplex™ STI Essential Assay Ct > 26). Results of the point-of-need LAMP workflow setup are shown in Fig 3.

Results obtained with the simplified protocol (point-of-need workflow) were compared with Allplex™ STI Essential Assay performed on the eluates obtained by automated extraction. Sensitivity, specificity, PPV, NPV, test accuracy and Cohen’s Kappa were separately calculated for every set of primers/probes based on the number of valid tests for every tested pathogen. Sensitivity ranged from 69.2% for *M*. *genitalium* to 92.6% with *U*. *parvum* primers, and specificity ranged from 96.2% for *U*. *parvum* to 100% for all other pathogens. PPV and NPV ranged from 96.2% for *U*. *parvum* to 100% for all other pathogens, and from 64.3% for *M*. *hominis* to 93.9% for *C*. *trachomatis*, respectively. Overall, test accuracy was above 89% for all pathogens. Calculated Cohen’s Kappa highlighted a good to really good agreement between the two workflows, ranging from 0.72 for *M*. *hominis* to 0.89 for *U*. *parvum*. Detailed information regarding test sensitivity, specificity, PPV, NPV, accuracy and Cohen’s Kappa for the simplified workflow are reported in Table 3.

**Table 3:** Results of the statistical analysis of the results obtained for point-of-need workflow setup. Data obtained from the analysis of samples processed with the gold standard workflow (extracted with STARMag 96 X 4 Universal Cartridge Kit and amplified with Allplex™ STI Essential Assay) were compared with data obtained from the analysis of the same samples with the point-of-need simplified workflow (extracted with a quick thermal/chemical extraction and amplified with Enbiotech STI Panel pathogen-specific primers/probe sets). For every indicator its respective 95% confidence interval was calculated.

	Sensitivity	Specificity	PPV	NPV	Accuracy	Cohen’s Kappa
***C*. *trachomatis***	82.4%	100.0%	100.0%	93.9%	95.2%	0.87
	56.57–96.20	92.29–100.00	76.84–100.00	83.13–98.72	86.71–99.01	0.73–1.00
***T*. *vaginalis***	77.8%	100.0%	100.0%	83.3%	89.5%	0.79
	39.99–97.19	69.15–100.00	59.04–100.00	51.59–97.91	66.86–98.70	0.51–1.00
***N*. *gonorrhoeae***	75.0%	100.0%	100.0%	85.7%	90.0%	0.78
	34.91–96.81	73.54–100.00	54.07–100.00	57.19–98.22	68.30–98.77	0.50–1.00
***M*. *genitalium***	69.2%	100.0%	100.0%	88.6%	90.9%	0.76
	38.57–90.91	88.78–100.00	66.37–100.00	73.26–96.80	78.33–97.47	0.54–0.98
***M*. *hominis***	87.8%	100.0%	100.0%	64.3%	90.0%	0.72
	73.80–95.92	66.37–100.00	90.26–100.00	35.14–87.24	78.19–96.67	0.50–0.94
***U*. *parvum***	92.6%	96.2%	96.2%	92.6%	94.3%	0.89
	75.71–99.09	80.36–99.90	80.36–99.90	75.71–99.09	84.34–98.82	0.76–1.00
***U*. *urealyticum***	86.8%	100.0%	100.00%	81.08%	91.57%	0.86
	74.66–94.52	88.43–100.00	92.29–100.00	64.84–92.04	83.39–96.54	0.71–1.00

CI = Confidence Interval, PPV = Positive Predictive Value, NPV = Negative Predictive Value.

## Discussion and conclusions

While standard nucleic acid amplification tests, specifically PCR-based assay, represent the gold standard for STIs diagnosis, due to their high sensitivity and specificity, they require trained personnel and expensive laboratory instruments, limiting the use of molecular methods to centralised laboratories. This, especially for STIs, clashes with the need of timely and near-of-patient results. In order to better fit the clinical application of STIs diagnosis, easy and rapid molecular tests run on portable instruments may represent a good compromise. In the recent years funding and expertise have been remarkably redirected to the development of novel molecular tests as an answer to the rise of STIs. More specifically, loop-mediated isothermal amplification-based tests have increasingly been employed in the last years for different pathogens [22], including SARS-CoV-2 [23], with promising results. In this study we evaluated the performance of a new real-time LAMP-based assay for the detection of seven bacterial and protozoan species responsible for STIs by analysing real clinical samples from individuals with suspected STIs following a quick thermal/chemical genomic DNA extraction, after which 60-minute amplification reactions were run isothermally. This assay, not requiring specialised laboratory technicians or sophisticated instrumentation to be carried out, is within the reach of outpatient settings, obstetrics, and gynaecology clinic, hence guaranteeing on-field access to early diagnosis [16, 24]. Enbiotech STI panel results were compared with Allplex™ STI Essential Assay (Seegene), the latter representing the gold standard high-throughput PCR test in many clinical microbiology laboratories.

The results obtained in this study showed a good diagnostic performance of the LAMP-based method and highlighted the potential of the essay to be deployed as a low-throughput test in clinical settings, allowing for a rapid, timely and accurate detection of STI-responsible pathogens: for every tested pathogen the point-of-need workflow showed a near perfect specificity despite a reduced sensitivity for low pathogen concentration samples. Overall test accuracy and concordance with the gold standard, as attested by Cohen’s Kappa, were nonetheless always good. During this study, some criticalities of the point-of-need LAMP workflow emerged, mainly derived from technical limitations of the simplified extraction method to deal with complex matrices, such as anal swabs, urine, and semen from which amplifiable DNA can be extracted and purified with difficulty. These results, although preliminary and needing a greater number of samples for further validation, suggest a great potential of this LAMP-based assay, which nonetheless needs additional development and implementation. Additional purification steps during genomic DNA extraction may decrease the number of false-negative and invalid results, facilitating the use of this test, with a certain degree of certainty about the reliability of the result, on a wider range of genital or extra-genital samples. Altogether, if false negative results (both due to a low pathogen load or to the inability of the point-of-need extraction method to successfully extract DNA from complex matrices) may lead to underdiagnosis of STIs, conversely, false positive results, recorded for more than one pathogen, may lead to an excessive and detrimental administration of unnecessary antibiotics. Both eventualities result disadvantageous in the context of rising STIs number, further encouraging an improvement of the diagnostic performance of the LAMP test. According to literature data, by means of a mathematical model, it has been demonstrated that a reduced sensitivity of rapid molecular tests may nonetheless assure a greater “diagnostic yield”, defined as a higher number on patients receiving a timely diagnosis and hence being treated accordingly, if compared to standard molecular tests, which can claim a higher sensitivity but whose overall diagnostic efficacy is hampered by longer waiting times (rapid test paradox): if a rapid test, although with a reduced sensitivity, can guarantee a sample-to-results time of minutes or hours, standard tests run in centralised laboratories, can in turn require days, or even up to one week, to produce a response to the clinical suspicion [25, 26]. In these cases, accessibility, jointly determined by rapidity, deliverability and user-friendliness may represent a good trade-off for diminished accuracy. As such molecular diagnostic tests capable of providing immediate results would facilitate to break the chains of transmission. Moreover, at this stage, testing a single sample requires setting up seven separate reactions, one with each designed set of primers/probe unless molecular testing is driven by a symptoms-based specific clinical suspicion capable of narrowing the spectrum of possibly responsible pathogens. Further implementation of multiplexing would certainly endure greater overall efficiency of the assay by reducing hands-on-time and turn-around-time, and thus speeding up responses for the clinician.

In conclusion and given the premises, this LAMP rapid test, despite some limitations is worthy of further refinement, and certainly may meet the needs of speed required by clinicians, representing a competitive alternative to other gold standard PCR-based (and laboratory-centric) assays, potentially meeting all the ASSURED criteria (Affordable, Sensitivity, Specificity, User friendly, Rapid, Equipment free, Delivered) published by World Health Organization’s Special Program for Research and Training in Tropical Diseases [27], thus harbouring a good potential to be integrated in a decentralized diagnostic system capable of timely diagnosis, guide treatment and inform disease control strategies and improve overall patients outcomes [28].
